# Indents

**Published:** 1908-08-15

**Authors:** 


### INDENTS.

☞ **Brief Articles of Technical or Scientific Value will receive notice and comment. Contributions invited**

**Tannic Acid for Pyorrhea Alveolaris.** In connection with pyorrhea alveolaris, neuralgic or rheumatic pains are sometimes met, which affect one or more teeth and yield to no remedy, so that the patients often ask to have the teeth extracted. In such cases a concentrated solution of tannic acid—that is, one part of tannic acid to five parts of spts. vini rect. (dilute alcohol)—should be applied. With this solution the edge of the gingivae is painted several times on the sensitive side, whereupon the pain will disappear. This solution generally acts better than silver nitrate or any other remedies. The teeth also become firmer.—M. KNER, *Wiener med. Presse.*

**Why it Adheres.** The following from the *Forum*, by Dr. A. E. Webster, gives in part the explanation for the adhesion of cement to surfaces etchable by phosphoric acid. It may be taken advantage of with profit in the setting of crowns and orthodontic appliances

Every cavity, whether for cements, inlays, or metal fillings, should be so formed that the filling cannot get out without either the fracture of the filling or the tooth. True, adhesiveness can hardly exist between two solids. But you ask me what holds a piece of cement on the labial surfaces of an incisor even after an orthodontia band has been removed; in fact, it has to be chiseled off. This is what happens in such cases: Phosphoric acid when applied to either dentine or enamel will immediately dissolve out the surface irregularly into eminences and depressions with many undercuts. When the cement is hardened, these pieces of cement in the undercuts must be broken off before the cement can be dislodged. Nothing of this kind happens when phosphoric acid is applied to either porcelain or gold, and thus we see the cement remain in the cavity and the inlay come away quite clean when an inlay fails. If cements are placed in a cavity with no excess of acid then there can be no etching of the tooth substance and consequently no so-called adhesion. To get the greatest attachment of a tooth to a tooth first apply some of the fluid for a few minutes or so and then wipe dry and apply the cement. This cannot be done in deep cavities because of the irritation of the pulp. There is no adhesion, but there is an inlocking of the particles of cement which must be broken before the cement can be dislodged.—*Office and Laboratory.*

**The Preparation and Use of Fluxes.** Borax glass has decided advantages over ordinary borax as a flux. There is no intumescence, hence little disturbance of solder or production of pits. Borax glass must, however, be used without the addition of water. The use of the powder dusted on is too inaccurate to insure good results.

I am indebted to a European publication for the suggestion of the use of vaselin as a vehicle for the application of any powdered fluxing agent. The vaselin burns without residue, leaving the powdered flux evenly distributed when applied.

For general use the following formula will be found practical and convenient:

| | | |
|---|---|---|
| Take— | White vaselin, - - - - | 2 parts: |
| | Powdered borax glass, - - - | 1 part; |
| | Chlorophyl, - - - - | q. s. to color. |

The borax glass must be reduced to the finest possible powder and incorporated with the vaselin when the latter is n a molten condition. The chlorophyl is added in small quantity to produce a pleasing color.

The compound is applied to the work before heating, with a fine sable brush, and the pieces of gold or silver solder are simply greased over with it. It should not be necessary to apply it after the piece is heated, as the vaselin melts and spreads the finely powdered flux into all joints and over surfaces to be covered.

Some care is necessary to avoid the use of an excess of flux, as well as to use the larger pieces of solder so that no borax is confined under them. Pits thus made will be found smaller than when common borax is used, and it will be noted that they are more nearly filled with the fused flux. With judgment in its use and a proper manipulation of the flame, perfect pieces of large size are easily produced.

For soft solder, composed of either tin or tin and lead, a saturated alcoholic solution of zinc chlorid crystals will be found superior to an aqueous solution or to the deliquescent chlorid.—A. R. COOKE, *Stomatologist.*

**Patience.** If I had the training of every boy in the land one of the first lessons I should try to teach him would be to overcome impatience. How long it does take the average individual to learn this lesson, and how seriously handicapped an individual is in life till he has it learned. The proverbial impetuosity of youth is all right to get up steam with, but it requires the balance wheel of patience to make the machine run true. Whenever I set any one growing impatient and fuming because things do not go to their liking, I am impressed with the awful waste of energy and dissipation of power. Calmness always controls, but impatience perverts. It is invariably the patient man who wins in the end. and who lives the longest.

If things do not work just right, keep cool. You will probably need all your reserve power to meet the situation without wasting it in impotent stamping. Battering yonr head against a brick wall hurts nothing so much as it does your head, and a moment's quiet reflection to steady your nerves is worth more than an hour's rampant rage.

One of the chief demands for patience is in dealing with the public. All sorts and conditions of temperament confront the individual who comes in daily contact with the rank and file of humanity. Impatience with the foibles of people soon leads to inharmony, inharmony to disagreement, disagreement to distrust, and distrust to hate. You simply cannot hold people if you are impatient with them. When anyone of a trying temperament comes into your business life study his peculiarities. and have patience. You can never control people unless you understand them, and you cannot understand them unless you study them. To do this in a way to search out the hidden springs of motive which impel their actions requires patience.

And the best of it all is that the cultivation of patience is one of the most effective means of self-development. You can never hope to control others unless you can first control yourself, and the very effort to control yourself constitutes growth. To be patient is to grow.

Lincoln was a patient man and so was Grant. Neither would have accomplished what he did—in fact, neither would have ever been heard of in the world—had it not been for this sublime quality. All really great men have been patient, though in some instances brief pericds of impetuosity have temporarily hidden the larger attribute. The patience of Job—just his patience—has made his name immortal. We cannot all be immortal in the sense that Job was, but we can all try to be patient, and that is the first step to immortality.

One of the best means of developing patience in dealing with people is to learn to look at the other man's point of view. Remember always that there are two sides to every question—sometimes many more than that—and if you put yourself in the other fellow's place and see the matter as he sees it, even if you do not agree with him, you are sure to be more patient with him.

If humanity would learn this lesson there would be fewer lawsuits, less contention, greater harmony and more certain happiness. Strive to be patient—even with fools. They may not know that they are fools.—C. N. Johnson in *Review.*

**Treatment of Pyorrhea.** The first consideration is to place, as far as possible, the teeth in their proper relation as to occlusion, correcting the interdental spaces and removing all sources of irritation at the margins of the gingivæ. This may require, in part, the services of the orthodontist, who should follow our treatment so as to give the teeth better positions, and in so doing reconstruct the alveolar processes.

The main consideration, however, is the removal of *all* deposits upon the teeth. If there be necrosed bone, curettement is necessary, or, if the apex of the root has become resorbed so as to form a needle-like extremity, amputation is desirable.

The first treatment, then, is surgical. This is a process of instrumentation which requires suitable instruments and delicacy of touch, skill, and an admirable patience. In fact, most of one's success lies in this procedure. It is definitely *the* treatment of pyorrhea alveolaris, because around these deposits upon the teeth are the foci of infection and inflammation which institute pyorrhea.

The second treatment is medicinal. Various remedies have been used to assist in the dislodgment of the deposits. Lactic acid has superseded all other remedies for this purpose. For some time I have been using a preparation made by Parke, Davis & Co. of Detroit, consisting of lactic acid and cresylic acid, ten per cent. of the latter being used in the mixture. The idea of incorporating the cresylic acid with the lactic acid is to impart to it a marked germicidal power. Cresylic acid is homologus with phenol or carbolic acid. It is a clear, colorless liquid of creosote-like odor and dissolves in alkaline solutions very readily. Its physiologic action is like that of carbolic acid, but not so toxic in its action, nor is it caustic when applied to tissues as is carbolic acid. Its germicidal power is greatly in excess of that of carbolic acid is about three times as powerful as pure phenol, and only one-third as active as a poison. Cresylic acid is the same as tricresol.

So far as used this remedy seems efficient, and I have not observed any difficulty in its employment.

There are many cases which do not require the use of lactic acid. In such cases, and always as a subsequent treatment to lactic acid, I have been using with much satisfaction sulfocarbolate of zinc. Indeed, this material has been my main reliance in all cases of pyorrhea alveolaris or gingivitis. I therefore desire to present sulfocarbolate of zinc as a medicament of great efficiency. Sulfocarbolate of zinc—or, as technically known, phenol-sulfonate of zinc —is obtained by heating a mixture of carbolic acid and sulfuric acid and saturating the product with zinc oxide, then evaporating and crystallizing. Sulfocarbolate of zinc (Merck) is readily obtained at a good pharmacy, and consists of colorless, transparent, and efflorescent crystals which are soluble in about twice their weight of water. From the ingredients of this material it is readily observed that it affords a compound that should prove valuable in the treatment of pyorrhea alveolaris and other phases of gingivitis. The U. S. Dispensatory says that "sulfocarbolate of zinc is an antiseptic, astringent, and stimulant to foul ulcers and in subacute inflammations of the mucous membrane."

Practical experience proves to me that sulfocarbolate of zinc contracts the gingivæ and tissues about a loosened tooth more rapidly than any other of the means employed. This is of decided advantage, for, in contracting the gingivæ, entrance to the pockets is prevented. which is extremely desirable. It is useful when the gums are swollen from crowns or bands. It is very excellent in hemorrhage, and fistulous tracts are closed by its use. Its employment involves no detriment to any of the tissues of the mouth. Being refrigerant, sulfocarbolate of zinc may cause some complaint because of its coldness, but warming the solution will modify this difficulty. For treatment, use ten per cent. of solfucarbolate of zinc dissolved in cinnamon water. To color this use tincture of cudbear. After the deposits have been removed and the pockets thoroughly cleaned with hydrogen dioxid and warm water, inject freely, but with not too much force, the solution of sulfocarbolate of zinc to completely overflow the pocket, then massage the tissues about the tooth quicklv. The Sub-Q syringe is an excellent instrument to use for injecting either the zinc or lactic acid. The barrel and piston of this syringe are of glass, and the packing is mineral wool.

Massaging the gum is very useful in promoting circulation. Patients are prone not to give this feature of self-treatment good attention. I instruct them to wrap a 6 x 6 inch antiseptic dental napkin about the forefinger, and, wetting it with the zinc solution, massage the tissues about the teeth. The crossing of the fibers composing the napkin makes a sufficiently coarse surface to give a better effect in massaging than the bare finger.

My experience with sulfocarbolate of zinc in pyorrhea has demonstrated that there is seldom a return of the pyorrhea, and that for an ordinary case the number of treatments necessary is small. However, one must keep in mind that treatment depends in the first place upon the removal of the exciting causes, and that the impossibility of doing this renders some cases incurable. Definite treatment, therefore, consists first of surgical interference; secondly, of medical application of remedies that will reduce the tissues to normal conditions. This may be accomplished in most cases by the skillful dental surgeon.—Dr. W. H. Whitslar in *Cosmos*.

